# Diverse action of repeated corticosterone treatment on synaptic transmission, neuronal plasticity, and morphology in superficial and deep layers of the rat motor cortex

**DOI:** 10.1007/s00424-017-2036-5

**Published:** 2017-07-27

**Authors:** Joanna Kula, Anna Gugula, Anna Blasiak, Bartosz Bobula, Joanna Danielewicz, Alan Kania, Grzegorz Tylko, Grzegorz Hess

**Affiliations:** 1 Institute of Zoology and Biomedical Research, Jagiellonian University, 30-387 Krakow, Gronostajowa 9, Poland; 2 Institute of Pharmacology, Polish Academy of Sciences, 31-343 Krakow, Smetna 12, Poland

**Keywords:** Brain slices, Dendritic spines, Pyramidal neurons, Stress, Synaptic plasticity, Joanna Kula and Anna Gugula contributed equally to this work.

## Abstract

One of the adverse effects of prolonged stress in rats is impaired performance of skilled reaching and walking tasks. The mechanisms that lead to these abnormalities are incompletely understood. Therefore, we compared the effects of twice daily repeated corticosterone injections for 7 days on miniature excitatory postsynaptic currents (mEPSCs), as well as on synaptic plasticity and morphology of layers II/III and V pyramidal neurons of the primary motor cortex (M1) of male Wistar rats. Corticosterone treatment resulted in increased frequency, but not amplitude, of mEPSCs in layer II/III neurons accompanied by increased complexity of the apical part of their dendritic tree, with no changes in the density of dendritic spines. The frequency and amplitude of mEPSCs as well as the parameters characterizing the complexity of the dendritic tree were not changed in layer V cells; however, their dendritic spine density was increased. While corticosterone treatment resulted in an increase in the amplitude of field potentials evoked in intralaminar connections within layer II/III, it did not influence field responses in layer V intralaminar connections, as well as the extent of chemically induced layer V long-term potentiation (chemLTP) by the application of tetraethylammonium (TEA, 25 mM). However, chemLTP induction in layer II/III was impaired in slices prepared from corticosterone-treated animals. These data indicate that repeated 7-day administration of exogenous corticosterone induces structural and functional plasticity in the M1, which occurs mainly in layer II/III pyramidal neurons. These findings shed light on potential sites of action and mechanisms underlying stress-induced impairment of motor functions.

## Introduction

The primary motor cortex (M1) contains cortical representations of movements [8, 32]. Rat M1 controls voluntary movements by integrating afferent inputs from sensory and motor systems and producing coordinated output signals which generate and control skilled behaviors like reaching for food [41, 50] or rung ladder walking [1, 31]. Organization of the local circuitry in the M1 is complex; however, connections within the M1 follow general systematic patterns (reviewed in [15, 20]). Several thalamocortical projections reach upper and lower layers of the M1 but layer II/III pyramidal neurons of the M1 receive information mainly from the ventral anterior and ventral lateral (VA/VL) thalamic nuclear complex [17, 20, 49]. Layer II/III of the M1 also receives corticocortical afferent inputs, including a prominent innervation from the primary somatosensory cortex. The main output from layer II/III cells is conveyed to other cortical areas and to local layer V neurons, which send axons to subcortical structures and the spinal cord (reviewed in [15, 20]). Both within layers II/III and V extensive local monosynaptic intralaminar connections relay excitatory activity at distances up to 1–1.5 mm [16]. Firing of layer II/III neurons occurs both during preparatory activity of layer V cells and during the execution of movements [17, 20]. Pyramidal neurons of layers II/III and V not only express different synaptic connections patterns but they also differ functionally, e.g., in the capacity for rhythmic synchronization of activity [47].

Learning of a motor skill is associated with reorganization of movement representations [21, 37]. The mechanism of skill learning-related plasticity involves activity-dependent, long-term potentiation (LTP)—like strengthening of excitatory synaptic connections within layer II/III of the M1 [38–40]. Long-term potentiation in M1 has been shown to be dependent both on NMDA receptors and voltage-dependent Ca^2+^ channels (VDCCs) [2, 6].

Learning skilled forelimb tasks has also been shown to induce structural plasticity including formation of new dendritic spines on layer V pyramidal neurons [51] and an increase in total dendritic length of these cells [13, 22]. Acquisition of motor skills has been shown to promote dendritic spine formation in layer V pyramidal cells of mouse M1 when it coincided with the physiological peak of plasma corticosterone level which fluctuates in a circadian manner [25]. However, stress and excessive corticosterone impair the movement accuracy and alter movement patterns in skilled reaching and walking tasks [18, 31]. Corticosterone, administered repeatedly for 10 days, has been reported to induce dendritic spine elimination and deficits in retention of previously acquired motor skills [27].

We have recently demonstrated that repeated corticosterone administration for 7 days resulted in an increase in the frequency of spontaneous excitatory postsynaptic currents (sEPSCs) recorded from layer II/III pyramidal neurons, which was not accompanied by changes in spontaneous inhibitory postsynaptic currents (sIPSCs) or the excitability of layer II/III pyramidal cells of rat M1 [23]. We also analyzed the density of dendritic spines on layer II/III pyramidal neurons but did not observe its change in corticosterone-treated animals [23]. Given that layers II/III and V pyramidal cells play different roles in receiving, integrating, and sending output from the M1, in the present study, we aimed to determine whether repeated corticosterone administration induces functional changes in M1 excitatory synaptic transmission in layer V pyramidal neurons, similar to those observed in layer II/III pyramidal cells [4, 23]. Moreover, we compared the amplitudes of field potentials evoked in intralaminar connections within layers II/III and V. We also assessed the potential of these connections to undergo synaptic plasticity using chemically induced long-term potentiation (chemLTP) by tetraethylammonium (TEA), which is a reliable model of VDCC-dependent synaptic plasticity induction within M1 acting through protein kinase A (PKA) and the extracellular signal-regulated kinase 1/2 (ERK1/2) cascade [14, 19]. Since the influence of corticosterone on neuronal morphology may be limited to certain neurons or to a specific part of the neuron (reviewed in [30]), the present study also compared overall dendritic morphology as well as the density of dendritic spines on layers II/III and V pyramidal neurons in the M1 between control and corticosterone-treated rats.

## Materials and methods

### Animals and treatment

Experimental procedures were approved by the Animal Care and Use Committee at the Jagiellonian University and were carried out in accordance with the European Community guidelines for the use of experimental animals and the national law. Male Wistar rats, aged 5–6 weeks at the beginning of the experiment, were housed in groups and maintained on a 12-h light/dark schedule (light on: 0800 hours–2000 hours). Standard food and tap water were available ad libitum. Corticosterone (TCI Chemicals), suspended in 1% Tween 80, was administered subcutaneously (dose = 10 mg/kg, volume = 2 ml/kg) twice daily for 7 days [53]. Control animals received the vehicle, but otherwise, they were handled identically and were investigated concurrently with corticosterone-treated rats. In total, 38 animals were used in the study.

### Preparation of brain slices

Brain slices were prepared 2 days after the last corticosterone administration. Rats were anesthetized with isoflurane (Aerrane, Baxter) and decapitated. Their brains were quickly removed and placed in ice-cold artificial cerebrospinal fluid (ACSF) containing (in mM): 130 NaCl, 5 KCl, 2.5 CaCl_2_, 1.3 MgSO_4_, 1.25 KH_2_PO_4_, 26 NaHCO_3_, and 10 D-glucose, and bubbled with the mixture of 95% O_2_–5% CO_2_. Coronal slices (thickness = 400 μm) containing a part of M1 were cut from one of the hemispheres between 3.8 and 1.7 mm rostral to bregma using a vibrating microtome (Leica VT1000). Slices were stored submerged in ACSF at 30 ± 0.5 °C.

### Whole-cell recording of spontaneous and miniature excitatory postsynaptic currents from pyramidal neurons

Individual slices were placed in the recording chamber mounted on the stage of the Zeiss Axio Examiner.D1 microscope and superfused at 3 ml/min with warm (32 ± 0.5 °C), modified ACSF of the following composition (in mM): 132 NaCl, 2 KCl, 1.25 KH_2_PO_4_, 26 NaHCO_3_, 1.3 MgSO_4_, 2.5 CaCl_2_, and 10 D-glucose, bubbled with the mixture of 95% O_2_–5% CO_2_. Recording micropipettes were pulled from borosilicate glass capillaries (Harvard Apparatus) using the Sutter Instrument P-1000 puller. The pipette solution contained (in mM): 130 K-gluconate, 5 NaCl, 0.3 CaCl_2_, 2 MgCl_2_, 10 HEPES, 5 Na_2_-ATP, 0.4 Na-GTP, 1 EGTA, and 0.1% biocytin (osmolarity = 300 mOsm, pH 7.3). Pipettes had open tip resistances of approx. 6 MΩ. Layers II/III and V pyramidal cells were sampled from sites located at least 2.5 mm lateral to the midline and approx. 0.3 or 1.1 mm, respectively, below the pial surface approx. 100 μm below the slice surface and were identified as described previously [46]. Signals were recorded using the SEC 05-X amplifier (NPI), filtered at 2 kHz and digitized at 20 kHz using Digidata 1440A interface and Clampex 10.4 software (Molecular Devices).

The firing characteristics of the recorded cells were assessed using intracellular injections of rectangular current pulses of increasing amplitude (duration = 400 ms) in the current clamp mode. For each cell, the relationship between injected current intensity and the number of action potentials was plotted. The gain was determined as a slope of the straight line fitted to experimental data. The threshold current (*I*_th_) was determined as a current extrapolated at zero firing rate [3].

Spontaneous EPSCs (sEPSCs) were recorded for 4 min from neurons which were voltage-clamped at −76 mV [23]. Then, the slices were superfused with ACSF containing 0.5 μM tetrodotoxin (TTX, Abcam). After confirming a lack of action potentials in response to depolarizing current pulses, miniature EPSCs (mEPSCs) were recorded for another 4 min. Data were accepted for the analysis when the access resistance ranged between 15 and 18 MΩ and remained stable (<25% change) during the recording. sEPSCs and mEPSCs were detected offline and analyzed using the automatic detection protocol (Mini Analysis software, Synaptosoft Inc.). The threshold amplitude for the detection of a single event was set at 5 pA. The initial analysis done automatically by the software using a number of search parameters was afterwards verified visually. The *t* test was used when appropriate to compare the mean frequency, mean amplitude, rise time, and decay time constant of events. Data without normal distribution and equal variance were tested using the Wilcoxon Signed Rank Test. The Kolmogorov-Smirnov test was employed to analyze cumulative distributions of events.

### Field potential recording and chemLTP induction

Individual slices obtained from a separate group of animals (control *n* = 5, corticosterone-treated *n* = 6) were placed in the interface-type recording chamber and superfused at 2.5 ml/min with warm (32 ± 0.5 °C) ACSF of the composition identical to that used in whole-cell experiments. Concentric bipolar platinum/stainless steel-stimulating electrodes (FHC) were placed approx. 0.3 or 1.1 mm below the cortical surface to activate fibers running within layers II/III or V, respectively [14]. Direct-current pulses (duration = 0.2 ms) were delivered at 0.033 Hz. Field potentials (FPs), were recorded approx. 0.5 mm from the stimulation sites, using glass micropipettes filled with ACSF (1–3 MΩ). FPs were amplified (Axoprobe 1A, Axon Instruments), A/D converted at 10 kHz and stored using Micro1401 interface and Signal 2 software (CED).

Stimulus–response curves for each slice were fitted to the data points with the Boltzmann equation: *V*_*i*_ = *V*_max_/(1 + exp((*u* − *u*_*h*_)/ − *S*), where *V*_max_ is the maximum FP amplitude, *u* is the stimulation intensity, *u*_*h*_ is the stimulation intensity evoking FP of half-maximum amplitude, and *S* is the factor proportional to the slope of the curve. The threshold stimulation was determined as the stimulus intensity necessary to evoke a field potential of approximately 0.1 mV in amplitude. Statistical analysis of FPs was carried out using the *t* test.

For the induction of chemLTP, the stimulation intensity was adjusted to evoke FPs of 30% of the maximum amplitude and slices were superfused for 15 min with ACSF containing 25 mM tetraethylammonium (TEA, Sigma-Aldrich) [19]. The amount of chemLTP was determined as an average increase in the amplitude of FPs, relative to baseline, after stabilization of responses (between 75 and 90 min after the end of TEA application). Statistical analysis of chemLTP was carried out using the Mann-Whitney *U* test.

### Assessment of dendritic morphology of pyramidal cells

Slices containing neurons filled with biocytin during whole-cell recordings were fixed for 24 h in 4% formaldehyde in PBS. After rinsing in PBS, slices were incubated with 0.3% Triton X-100 solution in PBS for 24 h and thereafter with 0.3% Triton X-100 and Cy3-conjugated ExtrAvidin (1:200, Sigma-Aldrich), washed, mounted on glass slides, and coverslipped with Vectashield containing DAPI (Vector Laboratories). Slices were examined under the Zeiss LSM510 META confocal microscope (Microimaging GmbH). Layer II/III cells were imaged using the 20×/0.8 Plan-Apochromat objective with 0.7× digital zoom and layer V neurons were imaged with 10×/0.30 EC Plan-Neofluar objective with 0.7× digital zoom. The step size in *z* plane was 1.7 or 6.344 μm for the 20×/0.8 and 10×/0.30 objectives, respectively. HeNe green laser with 543-nm excitation wavelength and 560 LP emission filter was used to visualize stained neurons. To assess complexity of the apical and basal dendritic tree of neurons, dendritic tracing and 3D Sholl analysis with a 20-μm step size was conducted in ImageJ (NIH) using the Simple Neurite Tracer plugin [28, 36]. Neurons with asymmetrical dendritic trees (due to possible truncation), were excluded from the tracing. Tracing data were then processed in L-Measure [42] to acquire the number of branches and bifurcations, total dendritic length and maximal branch order of dendrites. Unpaired *t* test for each data set was performed.

### Dendritic spines classification and analysis

After decapitation preceded by the isoflurane anesthesia, brains of a separate group of five corticosterone-treated and five control animals were quickly removed from the skull and rinsed in ice-cold ACSF. Blocks of the brain tissue containing the M1 were isolated and subjected to Golgi-Cox staining method, using FD Rapid GolgiStain™ Kit (FD Neurotechnologies), according to the attached protocol. Briefly, the tissue was immersed in the impregnation solution for 2 weeks (solutions A and B) and subsequently in solution C for 5 days (at room temperature in the dark). After this procedure, brains were cut into coronal slices (420-μm thick) using a vibrating microtome (Leica VT1000) and mounted on gelatin-coated glass slides. After rinsing in deionized water, sections were placed in solutions D and E. The reaction was stopped by repeating the rinsing step. Finally, slices were dehydrated in increasing concentrations of ethanol (50, 75, 95, and 100%), submerged in xylene and coverslipped with DPX Mountant for histology (Sigma-Aldrich).

Slices were examined using Zeiss AxioImager M2 light microscope equipped with Zeiss AxioCamHRm and a motorized specimen stage in *Z*-axis. For dendritic spine counting, neurons were recorded as bright-field images using the oil immersion 63×/1.4 or 40×/1.3 Plan-Apochromat objective. To meet the Nyquist criterion, images were recorded in *Z*-axis with a step of 0.275 and 0.375 μm, respectively, whereas *X*-*Y* resolution was defined by both the pixel size of the camera and the 1.5× magnification of the camera connector. All images were subjected to deconvolution by means of Huygens Professional Software (version 4.2, SVI), according to [33]. Briefly, the theoretical point spread functions (PSFs) were defined for both objectives, taking into account the *X*-*Y* and *X*-*Z* resolutions of the images and the 550-nm wavelength as the average for the spectrum of visible light. Then, the images were inverted and subjected to deconvolution using the Tikhonov-Miller algorithm with the wide field mode and a 30 signal/noise ratio for background estimation. The deconvolved images were finally inverted to their original bright field form and the number of dendritic spines was calculated.

The cells were identified as pyramidal neurons by the existence of spines on their dendrites and a characteristic single thick apical dendrite originating from a cone-like cell body and bifurcating towards the pial surface. Dendritic spine density was assessed independently for II- and III-order branches of the basal part of dendritic tree, as well as for II-, III-, and IV-order branches of the apical part. Spines were counted manually in ImageJ (NIH) on the basis of high-resolution z-stack images.

Morphology of the dendritic spine changes during the process of their development, what is strongly associated with maturation of the synapses they form. Thus, in the current study, spines were divided into four categories. For distinguishing thin spines from filopodia, criteria proposed by [7] were used. Elongated dendritic membrane protrusions with no head were classified as filopodia (dendritic spines precursors), whereas immature spines with thin necks and small heads were identified as thin spines. Mature dendritic spines consisting of short thin necks and large heads were classified as mushroom and thick spines with no necks and large heads as stubby. For each data set, *t* test was conducted. All data are presented as the mean ± SEM.

## Results

### The effects of corticosterone on field potentials

**Fig. 1:**
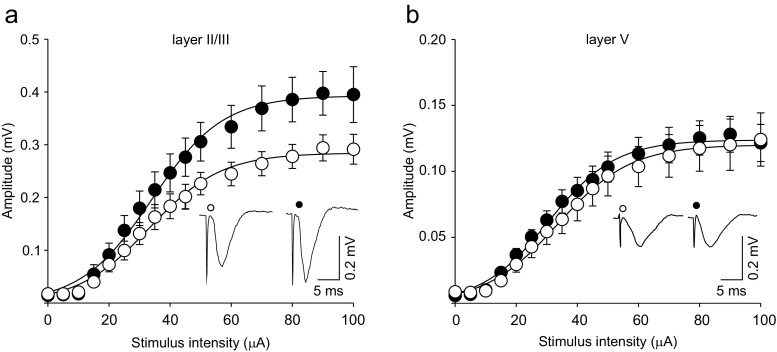
Corticosterone administration increases the amplitude of FPs in intralaminar connections within layer II/III but not layer V. Graphs show plots of the mean amplitude (±SEM) of FPs recorded in layer II/III (**a)** and V (**b**) in slices prepared from control (*open circles*, *n* = 11) and corticosterone-treated animals (*filled circles*, *n* = 15). *Continuous lines* represent the Boltzmann fits to the data. *Insets* show examples of FPs recorded during representative experiments at the stimulation intensity of 60 μA

The analysis of FPs evoked by stimulation of intralaminar connections within layer II/III revealed that the responses in slices obtained from corticosterone-treated rats were larger than in preparations from control animals over a wide range of stimulation intensities (Fig. 1a, Table 1). In contrast, amplitudes of FPs evoked in layer V did not differ between the experimental and control groups (Fig. 1b, Table 1).

**Table 1:** Effects of corticosterone treatment on parameters characterizing stimulus-response curves of FPs, calculated using the Boltzmann fits

Layer	Group	*V*_max_ (mV)	*u*_*h*_ (μA)	*S*	Number of slices
II/III	Tween	0.28 ± 0.02	33.21 ± 2.84	11.62 ± 0.79	11
	Corticosterone	0.39 ± 0.04**	34.02 ± 2.94	11.79 ± 0.73	15
V	Tween	0.12 ± 0.01	33.60 ± 1.62	11.90 ± 0.58	11
	Corticosterone	0.12 ± 0.03	30.53 ± 1.74	10.97 ± 0.57	15

*V*_*max*_ maximum field potential amplitude, *u*_*h*_ stimulation intensity evoking FPs of half-maximum amplitude, *S* factor proportional to the slope of the curve

**p < 0.01

### Corticosterone and neuronal excitability

**Fig. 2:**
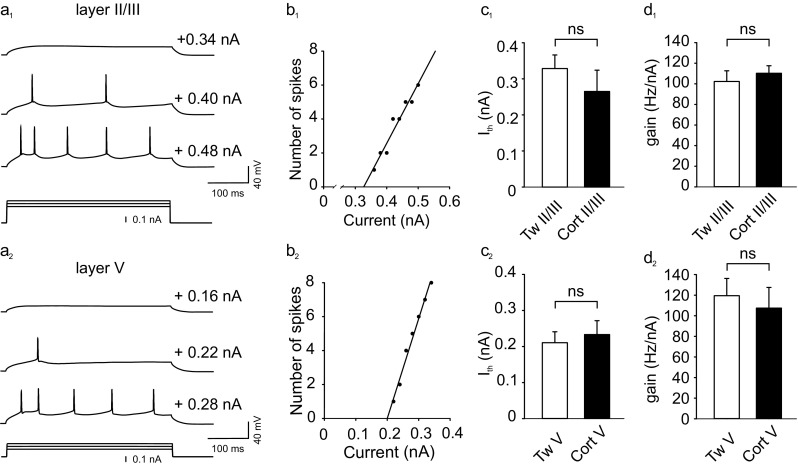
Corticosterone administration does not influence the intrinsic excitability of layer II/III and V pyramidal neurons. **a** Responses (*upper traces*) of a representative layer II/III neuron (**a**_**1**_) and layer V cell (**a**_**2**_) to sub- and suprathreshold depolarizing current pulses (*bottom trace*). **b** Graphs of the number of action potentials vs. the intensity of injected current for the cells shown in **a**_**1**_ (**b**_**1**_) and **a**_**2**_ (**b**_**2**_). The *slope of the straight line* fitted to experimental data represents the gain. **c** Mean (±SEM) extrapolated threshold current for layer II/III (**c**_**1**_) and layer V (**c**_**2**_) neurons in slices from control rats receiving Tween (Tw) and neurons originating from corticosterone (Cort)-treated animals. Note lack of significant differences (ns). **d** Mean (±SEM) gain for neurons from control and corticosterone-treated animals (labels as in **c**)

Whole-cell recordings were made from pyramidal neurons exhibiting a regular spiking firing pattern in response to a depolarizing current pulse (Fig. 2a_1_, a_2_). There were no statistically significant differences in the resting membrane potential and input resistance between neurons in slices originating from corticosterone-treated and control rats in both layer II/III (15 cells from 8 control and 17 cells from 11 corticosterone-treated animals) and layer V (14 cells from 8 control and 13 cells from 11 corticosterone-treated animals; Table 2). To assess intrinsic excitability, the relationship between the injected current and the firing rate was evaluated for each neuron (Fig. 2b_1_, b_2_). As illustrated in Fig. 2c, d, neither the mean gain nor the mean threshold current differed between experimental and control groups, indicating that corticosterone treatment did not influence the excitability of layers II/III or V pyramidal neurons.

**Table 2:** Basic parameters of recorded neurons

Layer	Group	*V*_*m*_ (mV)	*R*_*m*_ (MΩ)	*I*_th_ (nA)	Gain (Hz/nA)	Number of cells
II/III	Tween	−73.67 ± 2.00	43.12 ± 5.15	0.33 ± 0.04	102.29 ± 10.33	15
	Corticosterone	−73.94 ± 1.17	52.07 ± 4.36	0.26 ± 0.06	110.23 ± 7.33	17
V	Tween	−61.57 ± 2.51	55.30 ± 4.85	0.21 ± 0.03	119.4 ± 16.77	14
	Corticosterone	−60.38 ± 1.97	54.29 ± 6.48	0.23 ± 0.04	107.42 ± 20.06	13

Shown are means (± SEM). Differences between values for neurons from layers II/III and V are not significant

*V*_*m*_ resting membrane potential, *R*_*m*_ input resistance, *I*_*th*_ threshold current

### The effects of corticosterone treatment on spontaneous and miniature EPSCs

**Fig. 3:**
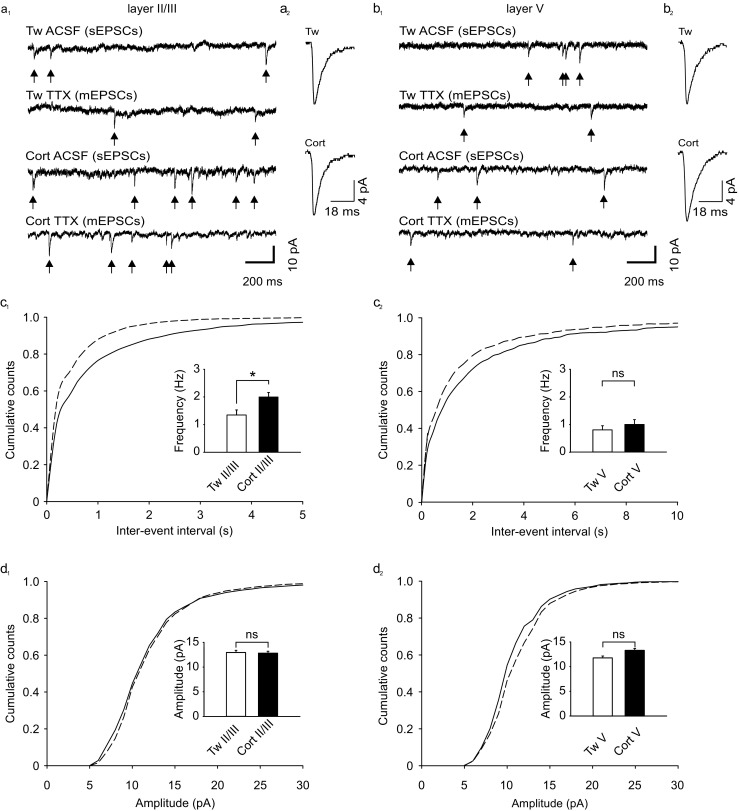
Corticosterone increases the frequency of mEPSCs in layer II/III but not in layer V pyramidal cells. **a**_**1**_ sample recordings from a representative layer II/III neuron in a slice prepared from control rat (*two upper traces*) receiving Tween (Tw) before (ACSF (sEPSCs)) and after addition of TTX to the slice incubation medium (TTX (mEPSCs)) and from a representative layer II/III neuron (*two lower traces*) from an animal receiving corticosterone (Cort) before (ACSF (sEPSCs)) and after addition of TTX to the slice incubation medium (TTX (mEPSCs)). **a**_**2**_ averaged mEPSCs recorded over a period of 4 min from a representative layer II/III neuron in slice prepared from animals receiving Tween (Tw) or corticosterone (Cort). **b**_**1**_, **b**_**2**_ Sample recordings from a representative layer V neuron in slice prepared from control rat and animal receiving corticosterone. (Labels as in **a**_**1**_, **a**_**2**_. **c**_**1**_–**d**_**2**_). Averaged cumulative histograms of inter-event intervals and amplitudes of mEPSCs recorded from neurons in slices prepared from animals receiving Tween (Tw, *solid lines*) and from pyramidal neurons from corticosterone-treated rats (Cort, *dashed lines*) in layer II/III (**c**_**1**_, **d**_**1**_) and V (**c**_**2**_, **d**_**2**_). Bar graphs in *insets* (**c**_**1**_ and **c**_**2**_) illustrate the mean (± SEM) frequency of mEPSCs. Bar graphs in *insets* (**d**_**1**_ and **d**_**2**_) illustrate the mean (± SEM) amplitude of mEPSCs. *White and black bars* represent control (Tw) and corticosterone-treated (Cort) groups. *p < 0.05; *ns* not significant. *Arrows* indicate events accepted for further analysis

To assess the contribution of spike-dependent synaptic transmission to the activity recorded from layers II/III and V pyramidal neurons, recordings were performed before and after addition of 0.5 μM TTX to ACSF (Fig. 3a_1_, b_1_). Exposure to TTX resulted in a slight but not significant decrease in the mean frequency of events recorded from layer II/III cells in slices prepared from control rats (before TTX: 1.51 ± 0.20 Hz vs. in TTX: 1.35 ± 0.18 Hz; *p* > 0.05, *Z* = −1931). The mean frequency of EPSCs in layer II/III neurons originating from corticosterone-treated rats was higher than EPSCs frequency recorded in neurons from control rats, but it also did not change significantly after exposure to TTX (before TTX: 2.21 ± 0.15 Hz vs. in TTX: 2.00 ± 0.16 Hz; *p* > 0.05, *t* = 1.692, df = 16). Exposure of slices to TTX did not affect the mean amplitude of recorded events either in the control group (before TTX: 13.95 ± 0.49 pA vs. in TTX: 12.95 ± 0.45 pA; *p* > 0.05, *t* = −1.911, df = 14) or in the corticosterone-treated group (before TTX: 13.68 ± 0.27 pA vs. in TTX: 13.00 ± 0.35 pA, *p* > 0.05, *t* = 2.11, df = 16).

In contrast to layer II/III, in layer V cells from control animals, the addition of TTX to ACSF resulted in a significant decrease in the mean frequency (before TTX: 1.39 ± 0.20 Hz vs. in TTX: 0.80 ± 0.15 Hz; *p* < 0.01, *Z* = −2919) and the mean amplitude of events (before TTX: 14.84 ± 1.07 pA vs. in TTX: 11.80 ± 0.38 pA; *p* < 0.001, *Z* = −3.296). Similarly, in layer V pyramidal neurons from animals receiving corticosterone treatment, addition of TTX to ACSF resulted in a decrease in both the mean frequency (before TTX: 1.41 ± 0.17 Hz vs. in TTX: 1.00 ± 0.18 Hz, *p* < 0.01, *t* = 3.70, df = 12) and the mean amplitude of events (before TTX: 14.31 ± 0.52 Hz vs. in TTX: 12.23 ± 0.37 Hz, *p* < 0.01, *t* = 4.08, df = 12). Thus, a significant part of the spontaneous synaptic activity recorded from layer V, but not layer II/III pyramidal neurons, was contributed by action potential-dependent neurotransmitter release.

Analysis of the parameters characterizing mEPSCs (recorded in the presence of TTX) revealed that corticosterone treatment caused an increase in the mean frequency of mEPSCs recorded from layer II/III pyramidal cells in comparison to control (2.00 ± 0.16 Hz vs. 1.35 ± 0.18 Hz, respectively; *p* < 0.05, *t* = −2.704, df = 30; Fig. 3c_1_; Table 3). However, there was no significant difference in the mean frequency of mEPSCs recorded from layer V cells between corticosterone-treated and control group (1.00 ± 0.18 Hz vs. 0.8 ± 0.1 Hz, respectively; *p* = 0.40, *t* = −0.849, df = 25; Fig. 3c_2_; Table 3). In line with these results, the difference between cumulative distributions of the inter-events intervals of mEPSCs was significant for layer II/III (*p* < 0.001; K-S test) but not for layer V neurons (*p* > 0.99). The mean amplitude of mEPSCs remained unaffected by corticosterone treatment in comparison to control both in layer II/III (13.00 ± 0.35 vs. 12.95 ± 0.45 pA, respectively; *p* = 0.93, *t* = −0.091, df = 30; Fig. 3c_1_; Table 3) and layer V cells (12.23 ± 0.36 vs. 11.80 ± 0.38 pA, respectively; *p* = 0.42, *t* = −0.812, df = 25; Fig. 3c_2_; Table 3). The analysis of cumulative distributions of mEPSC amplitudes did not reveal differences ether in layer II/III (*p* > 0.99) or V neurons (*p* = 0.96). The rise time and the decay time constant of averaged mEPSCs were also similar in both groups and both layers (Table 3).

**Table 3:** Effects of corticosterone treatment on parameters characterizing mEPSCs

Layer	Group	Mean frequency (Hz)	Mean amplitude (pA)	Rise time (ms)	Decay time constant (*τ*, ms)	Number of cells
II/III	Tween	1.35 ± 0.18	12.95 ± 0.45	2.67 ± 0.01	9.72 ± 0.73	15
	Corticosterone	2.00 ± 0.16 *	12.82 ± 0.38	2.75 ± 0.12	8.25 ± 0.75	17
V	Tween	0.80 ± 0.15	11.80 ± 0.38	3.07 ± 0.17	8.56 ± 0.47	14
	Corticosterone	1.00 ± 0.18	12.23 ± 0.36	2.85 ± 0.09	8.19 ± 0.39	13

Shown are means (± SEM)

*p < 0.05

### The effects of corticosterone on the induction of chemLTP

**Fig. 4:**
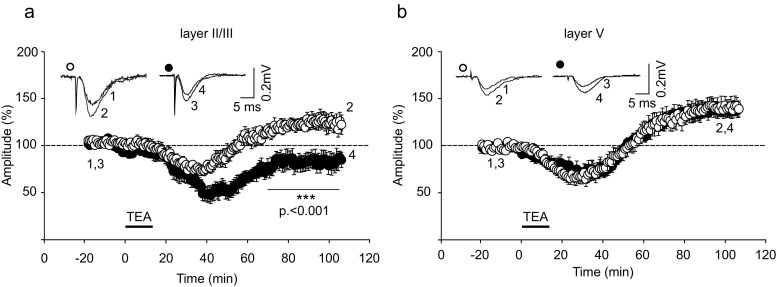
Corticosterone impairs the induction of chemLTP by tetraethylammonium (TEA) in layer II/III (**a**) but not in layer V (**b**). Plots show the amplitude of FPs (mean ± SEM) recorded from layer II/III in slices obtained from control rats (*white circles*) and from corticosterone-treated rats (*black circles*). Time of TEA application is indicated by a *horizontal thick line*. *Insets* show examples of FPs recorded at times indicated by numbers, before (*1* and *3*) and after (*2* and *4*) TEA application. ***p < 0.001; Mann-Whitney *U* test

In slices prepared from control rats, addition of the potassium channel blocker TEA (25 mM) to ACSF for 15 min induced chemLTP both in layers II/III and V (Fig. 4a, b). In control preparations, the mean amplitude of layer II/III FPs measured between 75 and 90 min after the end of TEA application increased to 121.61 ± 2.7% of the baseline. However, in slices from corticosterone-treated animals, no chemLTP occurred in layer II/III but the amplitude of FPs decreased below baseline (81.39 ± 3.8%, *p* < 0.001; Fig. 4a). In contrast, in layer V of the same slices, TEA-induced chemLTP of a similar magnitude in both control and corticosterone-treated group (135.15 ± 1.9% vs. 133.34 ± 2.04%, respectively; Fig. 4b).

### Corticosterone and dendritic tree morphology

**Fig. 5:**
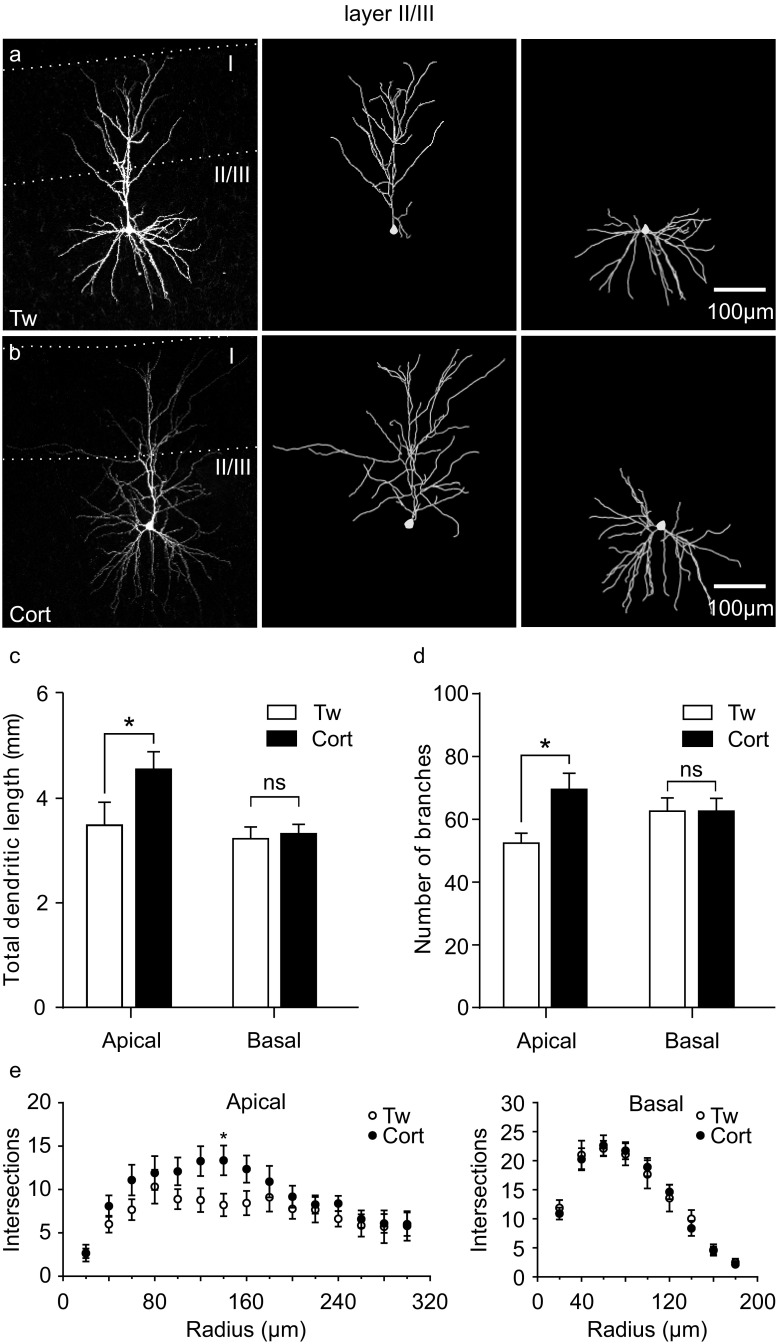
Repeated corticosterone treatment induces apical dendritic reorganization in II/III layer M1 pyramidal neurons. **a** Microscopic image of a representative, biocytin-filled layer II/III M1 pyramidal neuron of a control rat receiving Tween (Tw; *left*) and the 3D tracing of the same neuron’s apical (*middle*), and basal (*right*) dendritic tree. **b** Microscopic image of a biocytin-filled layer II/III M pyramidal neuron from an animal receiving corticosterone (Cort) (*left*) and the 3D tracing of the same neuron’s apical (*middle*) and basal (*right*) dendritic tree. **c** Bar graph of the mean (± SEM) dendritic length of layer II/III neurons showing a corticosterone-induced increase in length of the apical but not basal trees. **d** Bar graph of the mean number (± SEM) of the dendritic branching showing a corticosterone-induced increase in apical, but not basal trees. **e** Sholl analyses revealed a corticosterone-induced increase in the mean number (± SEM) of apical dendritic tree arborizations 140 μm from the cell body, and no effect of the treatment on the basal arborizations. *p > 0.05; *ns* not significant

Sholl analysis of the dendritic tree morphology was conducted on 9 layer II/III neurons from 8 control and 11 layer II/III neurons from 11 corticosterone-treated animals. Corticosterone treatment resulted in an increase in total dendritic length in the apical part of the dendritic tree (4560 ± 331.6 vs. 3488 ± 439.2 μm in control preparations; *p* < 0.05, Fig. 5c), an increase in the number of apical dendritic branches (69 ± 5.1 vs. 52 ± 3.15, respectively; *p* = 0.015, Fig. 5d), as well as in the number of bifurcations (34 ± 2.5 vs. 25.44 ± 1.6, respectively; *p* = 0.014). A tendency for an increase in the number of dendritic processes intersecting each concentric cycle was visible between 100 and 160 μm from the soma, and reached statistical significance at 150 μm (Fig. 5e). The apical tufts of layer II/III M1 neurons were not affected by the corticosterone treatment. In the basal part of the dendritic tree of layer II/III cells, no significant differences in the number of branches (62.1 ± 4.1 vs. 62.1 ± 4.2, respectively; *p* = 0.997), bifurcations (28.0 ± 2.0 vs. 28.1 ± 2.0, respectively; *p* = 2.04), total dendritic length (3326 ± 177.1 vs. 3230 ± 223.4 μm, respectively; *p* = 0.86), or dendritic tree geometry were detected (Fig. 5c–e).

**Fig. 6:**
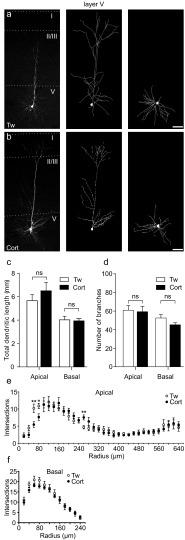
Repeated corticosterone treatment does not change the dendritic organization of layer V pyramidal neurons. **a** Microscopic image of a representative, biocytin-filled layer V pyramidal neuron of a control rat receiving Tween (Tw; *left*) and the 3D tracing of the same neuron’s apical (*middle*) and basal (*right*) dendritic tree. **b** Microscopic image of a biocytin-filled layer V M1 pyramidal neuron from an animal receiving corticosterone (Cort; *left*) and the 3D tracing of the same neuron’s apical (*middle*) and basal (*right*) dendritic tree. **c** Bar graph of the mean (± SEM) dendritic length of the layer V M1 neurons showing no effect of corticosterone on either apical or basal dendritic trees. **d** Bar graph of the mean number (± SEM) of dendritic branches showing lack of corticosterone-induced changes in either apical or basal trees. **e** Sholl analyses revealed that corticosterone induced a local decrease in the mean number (± SEM) of apical dendritic tree arborizations 60–80 μm from the cell body, and no effect of the treatment on the basal arborizations. *p > 0.05; *ns* not significant

In contrast to layer II/III, treatment with corticosterone only slightly altered the geometry of layer V pyramidal neurons (11 cells from 7 corticosterone-treated animals and 9 cells from 4 control rats). These pyramidal neurons exhibited stereotypical dendritic morphology and prominent apical dendrite, indicative of thick-tufted corticofugal pyramidal neurons [48]. On the limited length of the proximal part, the complexity decreased, but it increased in the middle part of the apical dendritic tree (Fig. 6e). The differences between other dendritic tree parameters including total dendritic length (apical: 6509 ± 712.6 vs. 5659 ± 521.7 μm, respectively; *p* = 0.37; basal: 3933 ± 195.3 vs. 4024 ± 310.9 μm, respectively; *p* = 0.8; Fig. 5c), number of branches (apical: 58.45 ± 5.97 vs. 60.11 ± 4.98, respectively; *p* = 0.84; basal: 44.54 ± 2.17 vs. 51.78 ± 3.51, respectively; *p* = 0.08; Fig. 5d) and bifurcations (apical: 34.27 ± 3.87 vs. 31.44 ± 2.63, respectively; *p* = 0.57; basal: 21.08 ± 1.43 vs. 24.44 ± 1.8, respectively; *p* = 0.16) were not significant.

### Corticosterone and dendritic spine density

**Fig. 7:**
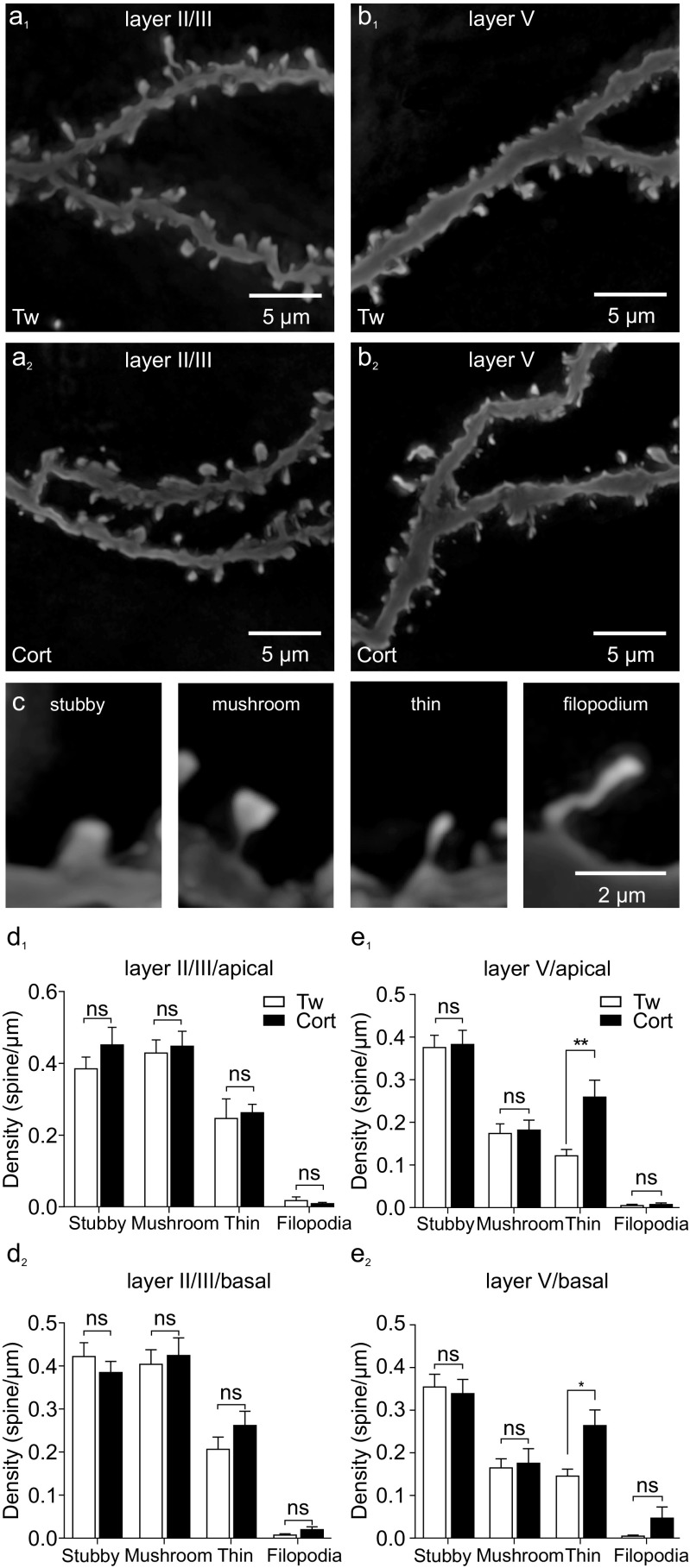
Repeated corticosterone treatment does not change dendritic spine density in layer II/III pyramidal cells but significantly influences spine density in layer V neurons. **a** Representative images of Golgi-Cox stained dendritic segments of layer II/III pyramidal neurons from control (**a**_**1**_) and corticosterone-treated rats (**a**_**2**_). **b** Representative images of Golgi-Cox stained dendritic segments of layer V pyramidal neurons from control (**b**_**1**_) and corticosterone-treated rats (**b**_**2**_). **c** Demonstrative images of dendritic spines segregated into four subclasses: stubby, mushroom, thin, and filopodia. **d** Bar graphs illustrating a lack of influence of corticosterone treatment on the mean number (± SEM) of distinguished spines subclasses on apical (**d**_**1**_) and basal (**d**_**2**_) dendrites in layer II/III cells. **e** Bar graphs illustrating an increase in the mean number (± SEM) of thin dendritic spines in corticosterone-treated rats on both apical (**e**_**1**_) and basal (**e**_**2**_) dendrites and lack of changes in remaining dendritic spines types in layer V M1. *p > 0.05; *ns* not significant

Dendritic spines were counted on randomly selected segments of the second-, third-, and fourth-order dendritic branches of the basal and apical dendrites of pyramidal cells. Spines were counted on 76 segments of 14 layer II/III neurons of the corticosterone-treated group (total length of analyzed dendrites = 3079 μm) and on 76 segments of 16 layer II/III neurons of the control group (total length of analyzed dendrites = 3991.9 μm). Consistently with our previous report [23], the calculated average total dendritic spine density did not differ significantly between the corticosterone-treated and control group either in the apical (1.1 ± 0.1 vs. 1.0 ± 0.1 spines/μm, respectively, *p* = 0.38) or in the basal part of the dendritic tree (total: 1.0 ± 0.05 vs. 1.0 ± 0.1 spines/μm, respectively, *p* = 0.84). The density of each morphological spine type investigated (Fig. 7c) was also similar both in the apical (stubby: 0.4 ± 0.05 vs. 0.4 ± 0.03 spines/μm, respectively, *p* = 0.31; mushroom: 0.4 ± 0.04 vs. 0.4 ± 0.04 spines/μm, respectively, *p* = 0.74; thin: 0.3 ± 0.02 vs. 0.2 ± 0.05 spines/μm, respectively, *p* = 0.77; filopodia: 0.09 ± 0.003 vs. 0.02 ± 0.01 spines/μm, respectively, *p* = 0.39) and in the basal part of the dendritic tree of layer II/III cells (stubby: 0.4 ± 0.03 vs. 0.4 ± 0.03 spines/μm, respectively, *p* = 0.37; mushroom: 0.4 ± 0.04 vs. 0.4 ± 0.03 spines/μm, respectively, *p* = 0.70; thin: 0.2 ± 0.03 vs. 0.2 ± 0.03 spines/μm, respectively, *p* = 0.20; filopodia: 0.02 ± 0.006 vs. 0.007 ± 0.003 spines/μm, respectively, *p* = 0.07, Fig. 7a_1_, a_2_, d_1_, d_2_).

Dendritic spines were counted on 66 segments of 12 layer V neurons of the corticosterone-treated group (total length of analyzed dendrites = 3430.9 μm) and on 76 segments of 16 neurons of the control group (total length of analyzed dendrites = 3533.98 μm) Treatment with corticosterone increased spine density both in the apical (0.9 ± 0.1 vs. 0.7 ± 0.05 spines/μm, respectively, *p* = 0.04) and basal part of the dendritic tree of layer V cells (0.8 ± 0.05 vs. 0.6 ± 0.04 spines/μm, respectively, *p* = 0.007, Fig. 7b_1_, b_2_, e_2_, e_2_). Analysis of spine types revealed an increased number of thin spines in corticosterone-treated rats, compared to control ones, both on the apical (0.3 ± 0.04 vs. 0.1 ± 0.06 spines/μm, respectively, *p* = 0.0004) and basal (0.3 ± 0.03 vs. 0.1 ± 0.02 spines/μm, respectively, *p* = 0.003) dendrites of layer V neurons. No significant differences were observed in the density of stubby spines (apical: 0.4 ± 0.03 vs. 0.4 ± 0.03 spines/μm, respectively, *p* = 0.87, basal: 0.4 ± 0.03 vs. 0.4 ± 0.03 spines/μm, respectively, *p* = 0.48), mushroom spines (apical: 0.2 ± 0.03 vs. 0.2 ± 0.03 spines/μm, respectively, *p* = 0.82, basal: 0.2 ± 0.03 vs. 0.2 ± 0.02 spines/μm, respectively, *p* = 0.77) or filopodia (apical: 0.007 ± 0.003 vs. 0.005 ± 0.003, respectively, *p* = 0.54, basal: 0.007 ± 0.003 vs. 0.005 ± 0.003 spines/μm, respectively, *p* = 0.60).

## Discussion

In the present study, we demonstrate for the first time that short-term, repeated corticosterone administration induces layer-specific structural and functional modifications in the rat M1. The data extend and complete our earlier results [4, 23], providing details about the mechanisms of corticosterone action that may underlie stress-induced deficiencies in motor functions.

Jointly, observed changes include an enhancement of the excitatory input to layer II/III pyramidal cells, assessed both with the recordings of spontaneous EPSCs [4, 23] and miniature EPSCs (this study) with concomitant increase in the amplitude of FPs evoked in interlaminar, vertically oriented [4] and intralaminar, horizontally oriented pathways within layer II/III (this study) as well as an impairment of the potential for LTP induced in vertically-oriented connections [4] and chemically induced synaptic plasticity (chemLTP) in horizontally oriented connections in layer II/III (this study). Observed increase in the amplitude of layer II/III FPs appears to relate to an increased frequency of miniature EPSCs. Moreover, the present study demonstrates corticosterone-induced increase in the structural complexity of the apical part of the dendritic tree of layer II/III pyramidal neurons. Notably, these effects do not occur in layer V pyramidal neurons, suggestive of a differential influence of corticosterone on the excitatory synaptic transmission in superficial and deep layers of the M1.

The present study investigated the effects of corticosterone treatment on dendritic spine density in layer II/III neurons subjected to the Golgi-Cox impregnation procedure. The present results confirm earlier conclusions, based on observations employing biocytin-filled neurons, that treatment with corticosterone did not modify spine density of layer II/III pyramidal neurons [23]. However, the present data also show an increase in the number of apical dendritic branches, a greater total dendritic length, and an increase in the number of bifurcations in the middle apical part of the dendritic tree of layer II/III cells after corticosterone treatment. It was reported that oblique apical dendrites as well as the upper basal dendrites of layer II/III pyramidal neurons of the barrel cortex receive excitatory inputs from local sources [10, 34, reviewed in 43]. However, our study revealed no structural alternations in the basal part of the layer II/III pyramidal cells’ dendrites. To our knowledge, no specific functional role of the input restricted to oblique apical dendrites of layer II/III pyramidal neurons of the M1 was described. Morphological changes limited to the middle oblique apical dendritic tree, observed in our study, may indicate unique sensitivity of this portion of layer II/III neurons to corticosterone and may constitute an anatomical substrate of corticosterone-induced enhancement of intralaminar excitatory connections within layer II/III of the M1.

In the rat medial prefrontal cortex (mPFC), chronic stress suppresses glutamatergic transmission and induces a decrease in the expression level of glutamate receptors and synaptic proteins [52]. This causes shrinkage of the apical dendritic tree of pyramidal neurons, which has also been reported after 3 weeks of corticosterone treatment [30]. In contrast, chronic stress has been reported to increase apical dendritic arborization in the orbital frontal cortex of the rat [26], resembling the corticosterone-induced effect observed in the M1 in the present study. Since the density of dendritic spines in these cells remained unchanged, it appears that the increase in complexity of the apical part of the dendritic tree is accompanied by a larger absolute number of synaptic connections, which might underlie the observed increase in the frequency of mEPSCs. Since these recordings have been performed in the presence of TTX, the observed effect of corticosterone treatment is not related to a general increase in the network activity in the M1. Alternatively, if the newly formed synapses are inactive, as a certain time period is necessary after spine growth to observe glutamate receptor currents (reviewed in [11]), the observed increase in mEPSCs frequency may be a result of enhanced spontaneous release of glutamate quanta from presynaptic terminals in pre-existing synapses. A lack of changes in the protein levels of postsynaptic glutamatergic receptor subunits after corticosterone treatment [23], as well as unchanged amplitude and kinetic properties of mEPSCs are consistent with the latter possibility. In line with this hypothesis, present data indicate that observed increase in the number of dendritic spines in layer V cells is not accompanied by changes in the frequency of mEPSCs, suggesting that “corticosterone-induced” synapses are inactive, at least in layer V neurons. Moreover, the higher density of dendritic spines in layer V cells results from an increase in number of thin immature spines, unable to form functional synapses [9]. Besides a lack of change in synaptic transmission parameters, the general complexity of layer V pyramidal neurons dendritic tree remained unchanged after corticosterone treatment, in contrast to layer II/III pyramidal cells. Importantly, it has been reported that repeated corticosterone administration influences neither the volume nor the cell number in rat M1 [5].

A lack of the possibility of chemLTP induction by TEA in layer II/III of slices originating from corticosterone-treated rats suggests that corticosterone-induced changes in excitatory transmission engage mechanisms involved in synaptic plasticity. Previously, we have shown that TEA-induced chemLTP in the M1 represents an NMDA receptor-independent, but VDCCs-dependent, form of synaptic potentiation [19] requiring the activation of the extracellular signal-regulated kinase (ERK) 1/2 cascade [14]. In this respect, the mechanism of chemLTP in the M1 resembles high-frequency stimulation (HFS)-induced LTP in the hippocampus where ERK activation is also required for the full expression of LTP [12, 44]. Although the induction of chemLTP by TEA in the hippocampal CA1 area is independent on NMDA receptors [35], HFS-induced LTP and TEA-induced chemLTP share similar Ca^2+^-dependent intracellular mechanisms. A key role in both phenomena plays the activation and autophosphorylation of postsynaptic alpha calcium-calmodulin-dependent protein kinase II (α-CaMKII) [24] and pre- and postsynaptic protein kinase C (PKC) [35]. The involvement of these pathways in the effects exerted by corticosterone and stress on the M1 remains to be established.

Repeated corticosterone administration has been proposed as a preclinical rodent model of chronic stress (reviewed in [45]). Repeated daily corticosterone administration at a dose of 40 mg/kg, lasting 7 days, induces signs of depression-like behavior in the forced swimming test and these effects are strengthened after longer corticosterone treatments lasting 14 or 21 days [29]. It is likely that the effects observed in course of the present study result from a direct activation of glucocorticoid (GR) and/or mineralocorticoid receptors (MR), which are abundant in the M1. Blockade of GRs and MRs has been shown to ameliorate some motor impairments resulting from stress [18]. Little is known about the effects of corticosterone on the structure and function of M1 neurons but available data indicate that a single dose of corticosterone (15 mg/kg) enhances both dendritic spine formation and elimination rate in mouse M1 [26]. Repeated corticosterone administration lasting 10 days has been reported to result in a loss of cortical spines in the superficial layers of mouse M1 by approx. 10% [27]. The diverging outcomes of cited experiments and our study, in which we administered 20 mg/kg of corticosterone for 7 days, might be a result of the use of different experimental models. It should be noted that we have previously observed a similar increase in the frequency of sEPSCs in layer II/III pyramidal cells after corticosterone administration lasting 7 and 21 days [4].

Acquisition of a motor skill involves strengthening of excitatory synaptic connections within layer II/III of the M1 [39, 40], an effect resembling the results of repeated corticosterone administration ([4, 23], this study). However, acquisition of a skill has also been found to result in an increase in the dendritic length and branching, but not spine density, in layer V pyramidal cells of the M1, accompanied by a reduction in the spine density in layer III pyramidal neurons [22]. Thus, the effects of skill acquisition and corticosterone treatment differ. Nevertheless, corticosterone-induced lack of the potential of intralaminar connections within layer II/III of the M1 to undergo synaptic plasticity is likely to hamper the possibility of acquiring a new motor skill.

The present results, together with our previous study, which compared the effects of corticosterone treatment on excitatory and inhibitory transmission [23], indicate that elevated corticosterone levels result in a distortion of the balance between the glutamatergic and GABAergic systems in layer II/III of the rat M1. Local M1 circuitry expresses predominantly top-down organization where descending excitation from a “preamplifier-like” network of upper-layer neurons drives output neurons in lower layers [49]. Our findings indicate that excitatory intralaminar interactions within layer II/III in the brain of corticosterone-treated animals treated are enhanced but the inhibitory input to layer II/III neurons remains unchanged. Thus, enhanced excitatory transmission in upper layers will consequently spread wider horizontally and distort the precision of the organization of motor maps by generating stronger than normal excitatory input to layer V corticospinal neurons which are hardly influenced by corticosterone treatment. Stronger activity of corticospinal motor output will exert enhanced excitatory drive on spinal motoneurons, which might explain reduced skilled movement accuracy in reaching and walking and increased performance speed observed in stressed rats [31].
